# Ageing Behaviour of Al–Mg–Si Alloys After Cryogenic and Room Temperature Deformation

**DOI:** 10.3390/ma13030554

**Published:** 2020-01-23

**Authors:** Belinda Gruber, Florian Grabner, Werner Fragner, Alexander Schökel, Florian Spieckermann, Peter J. Uggowitzer, Stefan Pogatscher

**Affiliations:** 1 Chair of Nonferrous Metallurgy, Department Metallurgy, Montanuniversität Leoben, Franz-Josef-Straße 18, 8700 Leoben, Austria; belinda.gruber@unileoben.ac.at (B.G.);; 2 Christian Doppler Laboratory for Advanced Aluminum Alloys, Chair of Nonferrous Metallurgy, Montanuniversität Leoben, Franz-Josef Straße 18, 8700 Leoben, Austria; 3 LKR Light Metals Technologies Ranshofen, Austrian Institute of Technology, Lamprechtshausenerstr. 61, 5282 Ranshofen, Austria; 4 AMAG rolling GmbH, Postfach 32, 5282 Ranshofen, Austria; 5 Deutsches Elektronen Synchrotron (DESY), Notkestr. 85, 22607 Hamburg, Germany; 6 Chair of Materials Physics, Department Materials Science, Montanuniversität Leoben, Jahnstr. 12, 8700 Leoben, Austria; 7 Laboratory of Metal Physics and Technology, Department of Materials, ETH Zürich, Vladimir-Prelog-Weg 4, 8093 Zürich, Switzerland

**Keywords:** aluminium alloys, cryogenic temperature, ageing kinetics, dislocation density, bake hardening

## Abstract

The aim of this study is to investigate the effects of cryogenic and room temperature pre-deformation on subsequent artificial ageing of Al–Mg–Si alloys. Naturally aged and pre-aged samples were strained to 5%, 10% and 20% at RT (25 °C) and under liquid nitrogen, and artificially aged at 185 °C. Pre-deformation generally increases ageing kinetics for both the naturally aged and pre-aged alloys, which increase in proportion to the degree of pre-deformation, and which are slightly more pronounced for the cryogenic condition. The peak strength is constant, except for when a low degree of pre-deformation is used, in which case it is slightly reduced. Cryogenically deformed samples show an increased strength and hardness, compared to samples pre-deformed at RT, when subjected to an equal magnitude of strain. This difference is reduced during artificial ageing. Synchrotron measurements reveal that this behaviour can be linked to the greater dislocation density, which is not completely recovered even after prolonged ageing at 185 °C.

## 1. Introduction

In the automotive industry, Al–Mg–Si alloys are preferably used as lightweight material due to their advantageous mechanical properties, such as good corrosion resistance, weldability, and great strength [1,2]. However, a deficiency of these alloys is their limited ductility [3]. Recent developments show an improvement in their formability at sub-zero temperatures [4,5]. Schneider et al. [6] identified an increase in uniform elongation of 42% in alloy AA 6016 when the temperature was lowered to −196 °C. In light of this, industrial deep drawing processes can be carried out at cryogenic temperatures with the advantage of affording greater freedom when it comes to design.

In the automotive industry, during the manufacturing of outer sheet panels, an additional heat treatment is applied. This heat treatment is applied after the last deformation step (deep drawing) and simultaneous to the coat of paint drying and hardening and is therefore called paint bake hardening [7]. In recent decades, lower temperatures and shorter times have been preferred for economic reasons [2]. This work assumes a current achievable standard of heat treatment, lasting for 20 min at 185 °C. However, with this short heat treatment, the peak aged condition for Al–Mg–Si alloys cannot be reached [8]. Moreover, the components are stored at room temperature prior to the paint bake process, due to delivery or manufacturing procedures. This leads to natural ageing (NA), and afterwards to negative effects during subsequent artificial ageing [9]. To prevent the influence of prior natural ageing, different pre-treatments, such as pre-ageing, are used. Pre-ageing is a further heat treatment step directly after the solution annealing. With this heat treatment, NA during RT storage (25 °C) is suppressed for a certain period of time, and an acceleration of precipitation kinetics upon subsequent artificial ageing is induced [10,11].

A modification of artificial ageing kinetics can not only be achieved with heat treatment prior to RT storage, but also through pre-deformation [7,12]. Deformation after solution treatment can suppress NA, presumably due to the annihilation of quenched-in vacancies at dislocations, and may therefore accelerate artificial ageing [13,14]. Plastic deformation directly prior to artificial ageing leads to fewer coarser precipitates, which are formed predominantly at dislocation lines [15,16]. The induced dislocations provide nucleation sites for the β’’ phase, which changes and accelerates the precipitation kinetics [17,18]. Facilitated pipe diffusion along dislocations leads to shorter peak ageing times, to a faster growth, and also to a faster coarsening of the β’’ phase. [19,20].

In general, deformation always occurs between the delivery of the material and the bake hardening process, because of forming operations like deep drawing. The degree of deformation varies between different processes, and also within a manufactured part itself. These variations can lead to different ageing responses and different remaining strain hardening contributions, due to different dislocation densities in the final products.

The influence of RT and cryogenic pre-deformation for different degrees and delivery states (i.e., pre-aged and naturally aged) on the paint bake process have not been fully explored in the relevant literature. Moreover, a clarification on the effect that the decay of the dislocation density has upon the thermal load of a paint bake process for the above-mentioned cases is lacking. In this article, we illustrate the effect of RT and cryogenic pre-deformation on the artificial ageing response and on the alteration of the dislocation density.

## 2. Materials and Methods

All specimens were produced from sheet material with 1.5 mm thickness of alloy EN AW 6016, with a chemical composition of Mg (1.1 wt.-%), Si (0.35 wt.-%), Mn (0.08 wt.-%), Fe (0.15 wt.-%) and Al (balance). The alloy was tested in two different conditions, naturally aged (T4) and pre-aged (PA). Both conditions were solution annealed at 540 °C in a convection furnace (Nabertherm N15/65SHA, Lilienthal, Germany) and water quenched. Pre-ageing was executed at 100 °C for five hours. The specimens exhibit a recrystallised microstructure with a grain size of 36 ± 12 µm. All specimen were stored in a Memmert IPP 400 incubator (Memmert, Schwabach, Germany) at 25 °C for two weeks.

After the intermediate storage, the actual measurements were carried out in the following sequence: Initially, the samples were pre-deformed via straining at either RT or −196 °C and then again stored for two weeks at 25 °C. Subsequently, a heat treatment at 185 °C for different times was applied. Directly afterwards, hardness measurements and tensile tests were carried out at RT.

For the pre-deformation tensile test, samples were cut from the sheet material perpendicular to the rolling direction, with a width of 12.5 mm and a reduced section of 57 mm in accordance with ISO 6892-1 [21]. Pre-deformation was carried out on a Zwick/Roell Z100 tensile testing machine (Ulm, Germany), with a extensometer (Sandner 20-10 O, Sandner-Messtechnik, Biebesheim, Germany) for elongation determination. When the measurements were conducted at cryogenic temperatures (LN_2_), the reduced section of the tensile testing sample, as well as the extensometer, were kept in a Dewar filled with liquid nitrogen. Previous measurements with this setup are explained in detail by Gruber et al. [22]. After pre-straining and two weeks of RT storage, the samples were artificially aged for different durations. The ageing treatment was carried out in an oil bath at 185 °C (Lauda Proline P 26, Lauda-Koenigshofen, Germany). The artificial ageing hardness curves were measured on an EMCO-TEST M4C hardness-measuring device (EMCO-TEST, Kuchl, Austria). The hardness testing was performed with the Brinell method HBW 2.5/62.5 in accordance with ISO 6506-1 [23]. Additionally, after 0, 10, 20 and 30 min of heat treatment at 185 °C, tensile tests were conducted with a strain rate of 0.008 s^−1^ and a gauge length of 50 mm in accordance with ISO 6892-1 [21]. Due to the pre-deformation, the samples were already in the shape of tensile testing samples, and also the direction of the load stayed the same. At least three measurements were accomplished for every hardness and tensile test. The hardness values represent the average of the measurements, with the standard deviation displayed through error bars and a representative curve for each condition is shown in the tensile test results.

Additionally, synchrotron measurements were conducted using a fixed photon energy of 60 keV on the Petra III beamline P02.1 at DESY (Deutsches Elektronen-Synchrotron, Hamburg, Germany). Diffraction patterns with a beam size of 200 × 200 µm were recorded by a 2-dimensional Perkin-Elmer XRD1621 detector (PerkinElmer, Waltham, Massachusetts, USA). The wavelength and sample to detector distance was calibrated using LaB6 (NIST660b, National Institute of Standards and Technology, Gaithersburg, MD, USA) and CeO_2_ (NIST674b, National Institute of Standards and Technology, Gaithersburg, MD, USA) standards, respectively. The samples were also cut from the pre-deformed samples and stored at 25 °C for two weeks prior to the measurements. To evaluate the dislocation density independence of the heat treatment, the samples were measured in situ during heating from 25 to 185 °C, and during the 20 min treatment at 185 °C. Afterwards the diffraction patterns with respect to the azimuthal range were integrated using pyFAI software (European Synchrotron Radiation Facility, Grenoble, France) [24]. The change in dislocation density was calculated by X-ray line profile analysis using Fityk (Marcin Wojdyr, Cambridge, UK) [25] for the peak position evaluation and Convolutional Multiple Whole Profile fitting (CWMP) (Gábor Ribárik, Budapest, Hungary) [26], with a combinatorial approach to achieve a high numerical stability [27,28].

## 3. Results and Discussion

**Figure 1:**
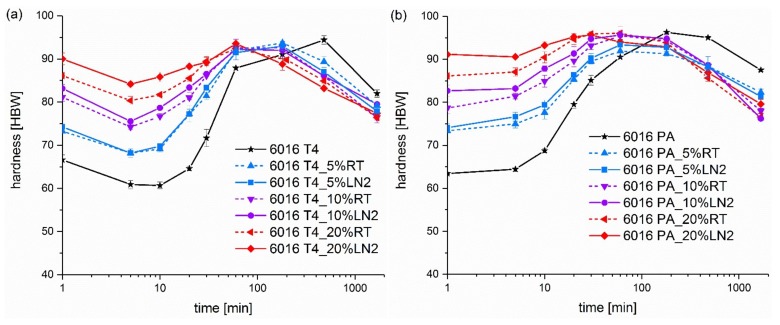
Ageing kinetics of alloy (**a**) EN AW 6016 naturally aged (T4) and (**b**) EN AW 6016 pre-aged (PA) at 185 °C after 0%, 5%, 10% and 20% deformation at 25 °C (RT, solid lines) and −196 °C (LN_2_, dashed lines).

Figure 1 displays the ageing kinetics at 185 °C of NA and PA alloy EN AW 6016. The solid lines represent pre-straining at −196 °C and the dashed lines represent pre-straining at RT. As a reference, also the ageing progression of the undeformed material is shown (in black).

In Figure 1a, alloy EN AW 6016 T4 exhibits a reduction in hardness in the first minute of artificial ageing, due to the dissolution of clusters formed from natural ageing. Significant re-hardening occurs by the formation of the β’’ precipitates, which is slow in comparison to solution-annealed or PA samples [9]. The same regression also occurs in the RT and LN_2_ pre-strained samples. In contrast, an increase in hardness at the beginning of the artificial ageing treatment can be achieved in the PA condition in Figure 1b. During the pre-ageing heat treatment, the first nuclei for the artificial ageing are formed, and thus, a formation of natural ageing phases during RT storage is suppressed for a certain time, leading to a more pronounced artificial ageing response [10,11].

With greater pre-straining, the initial hardness increases for both conditions. Simultaneously, the ageing kinetics accelerate and the peak hardness appears earlier. Pre-straining at RT promotes the nucleation of β’’, and also leads to a faster coarsening of the precipitates and to a decrease in hardness [13,17]. The dislocations from pre-straining are assumed to provide nucleation sites for the β’’ phase, and therefore to accelerate the artificial ageing kinetics [7,16].

Moreover, prior to the heat treatment, all hardness values of the LN_2_-deformed samples are greater than those of the RT deformed samples. This results from the higher strain hardening rate at lower temperatures in aluminium and its alloys [29]. Subsequently, samples deformed at lower temperatures have greater dislocation densities and thus exhibit greater strength and hardness values, despite the same amount of pre-straining [6,30]. Figure 1 shows that not only are the initial values elevated, but also the artificial ageing curves up to 30 min are shifted to greater values, due to LN_2_ pre-straining. Two potentially overlapping effects can be assumed: (i) The dislocation densities are greater at the beginning of the heat treatment and remain so, to a certain extent, for up to 30 min at 185 °C, despite an increased recovery rate [22]; (ii) The ageing behaviour changes due to cluster formation at dislocations [15,16,18,19,20] and is even more accelerated due to greater dislocation density.

It can be observed from Figure 1 that the time till peak hardness is reached is significantly reduced via pre-straining, while increasing pre-straining changes the maximum hardness only slightly over the course of the heat treatment. This especially applies to 20% pre-deformation. However, in Figure 1b, a 5% pre-straining at LN_2_ resulted in reduced maximum hardness, while still accelerating kinetics.

**Figure 2:**
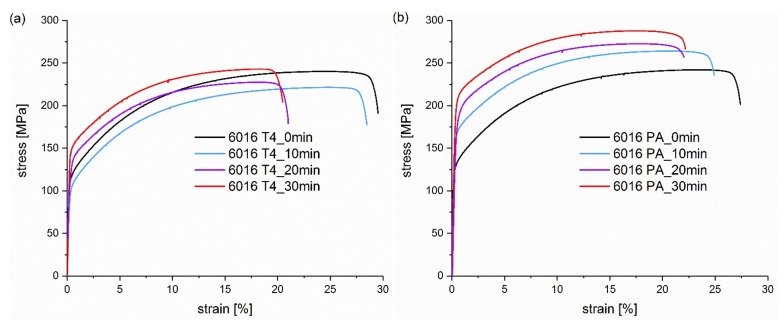
Tensile testing curves of alloy (**a**) EN AW 6016 T4 and alloy (**b**) EN AW 6016 PA heat treatment times from 0 min to 30 min at 185 °C.

**Figure 3:**
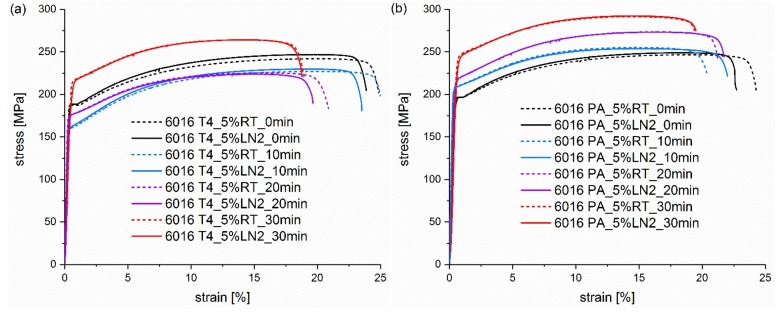
Tensile testing curves of alloy (**a**) EN AW 6016 T4 and alloy (**b**) EN AW 6016 PA pre-deformed for 5% at RT (dashed curves) and LN_2_ (solid curves). The curves in the individual diagrams result from different pre-deformation temperatures and heat treatment times from 0 min to 30 min at 185 °C.

**Figure 4:**
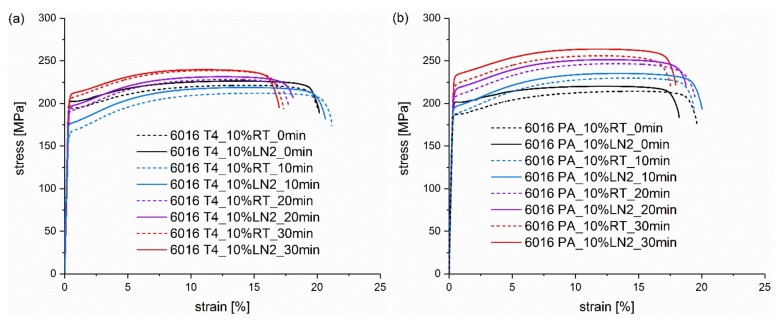
Tensile testing curves of alloy (**a**) EN AW 6016 T4 and alloy (**b**) EN AW 6016 PA pre-deformed for 10% at RT (dashed curves) and LN_2_ (solid curves). The curves in the individual diagrams result from different pre-deformation temperatures and heat treatment times from 0 min to 30 min at 185 °C.

**Figure 5:**
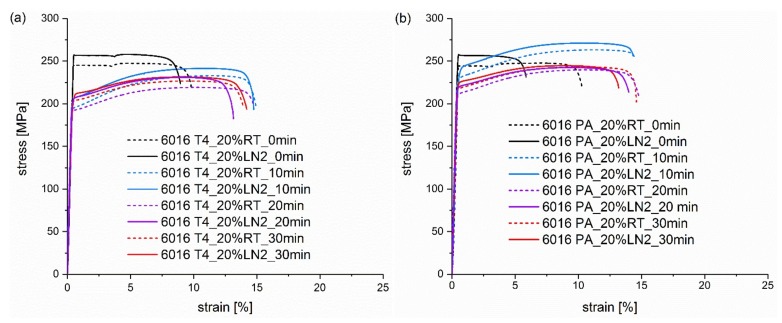
Tensile testing curves of alloy (**a**) EN AW 6016 T4 and alloy (**b**) EN AW 6016 PA pre-deformed for 20% at RT (dashed curves) and LN_2_ (solid curves). The curves in the individual diagrams result from different pre-deformation temperatures and heat treatment times from 0 to 30 min at 185 °C.

In order to gain an overview of the strength level during the paint bake hardening, tensile tests were conducted after 10, 20 and 30 min at 185 °C, and also at 0 min for the condition without heat treatment. In Figure 2, Figure 3, Figure 4 and Figure 5 the resulting curves of the different artificial ageing times are compared and separated in diagrams by initial natural aged (T4) or pre-aged (PA) condition and different degrees of deformation. Figure 2 displays the artificially aged tensile testing curves of undeformed samples, in order to obtain an overview of the ageing potential of the first 30 min between the natural aged and pre-aged sample. As already seen in the hardness curves, an initial decrease in strength (yield strength and ultimate tensile strength) occurs in the T4 condition, which recovers upon further heat treatment. In contrast, the strength of the PA sample continuously increases with the artificial ageing duration.

In Figure 3a,b, 5% of pre-deformation at RT and LN_2_ leads to an overlap of the tensile testing curves. No significant difference in strength with the pre-deformation temperature can be seen directly after straining and at heat treatment times from 10 to 30 min. Obviously, the pre-deformation is too low to result in noticeable differences between RT and LN_2_. Please note that the tensile samples were stored at RT for two weeks prior to the paint bake treatment, which has been shown to already reduce increased dislocation density created via deformation in cryogenic conditions [22]. This can explain the small difference between RT and LN_2_ yield strength before heat treatment, despite the increased strain hardening at low temperature pre-deformation. In addition to the gain in yield strength, the only indication of pre-deformation is the flow onset, especially in the not heat-treated samples. Unaffected by previous deformation temperature, this is expressed by a small plateau at the beginning of the flow onset, which disappears during the paint bake process. The stronger the applied pre-deformation, the more pronounced this plateau is and the longer the heat treatment is necessary to restore an original flow onset. This is the result of simultaneous processes during further deformation: the competition between strain hardening and softening due to dynamic recovery [22]. The flow onset is scarcely discussed in previous publications on RT pre-deformation, as most studies do not show tensile test curves. Mostly only hardness measurements or yield strength values are given [12,17,18]. Tensile test curves are usually displayed insufficiently to detect the flow onset behaviour, due to dynamic recovery, or for pre-deformations [31] which are too small to obtain a pronounced area signifying the start of flow, or for a heat treatment having been already carried out after the deformation [19,20], or for the plateau of the flow onset having already recovered.

At 10% pre-deformation in Figure 4a,b, a significant effect of the deformation temperature is visible, and the LN_2_ pre-strained samples exhibit tensile test curves that have shifted to greater values. As mentioned above, the initial variation is due to a greater strain hardening rate at lower temperatures [29] and the consequent increase of strength, despite the same degree of pre-stretching. This difference quickly diminishes during the heat treatment in the natural aged condition, whereas it is maintained slightly longer in the EN AW 6016 PA samples in Figure 4b. Even after 20–30 min, a difference of 10–15 MPa is still noticeable in the pre-aged condition, while the naturally aged sample only achieves a difference of ~8 MPa.

As expected, all samples in the naturally aged T4 condition show, at the beginning of the heat treatment, a decrease in strength because of cluster dissolution and the negative effect of natural ageing (see Figure 2a, Figure 3a and Figure 4a). However, the strength in the 20% pre-deformed samples decreases in the first 20 min at 185 °C also in the pre-aged condition, as displayed in Figure 5b. This can be attributed to the pronounced recovery at the beginning of the heat treatment causing a stronger impact than artificial ageing. Again, the LN_2_ deformed samples show a greater strength at the beginning, which deteriorates during the paint bake process but is still present after 30 min.

Deformation under LN_2_ is characterized by a greater work hardening than at RT, and thus, greater strengths are achieved with the same degree of deformation, as explained previously. At 20% strain, a strength level has been already reached which exceeds the ultimate tensile strength achieved at RT. Therefore, in the case of direct subsequent deformation, early failure occurs (Figure 5), as reported in a previous study [22]. After the heat treatment, these samples have a similar ultimate elongation compared to the RT samples and no damage is done to the material due to the cryogenic deformation results. This can be explained by the recovery already occurring during ageing, and thus, strain hardening wins in the battle with dynamic recovery during the tensile test.

**Figure 6:**
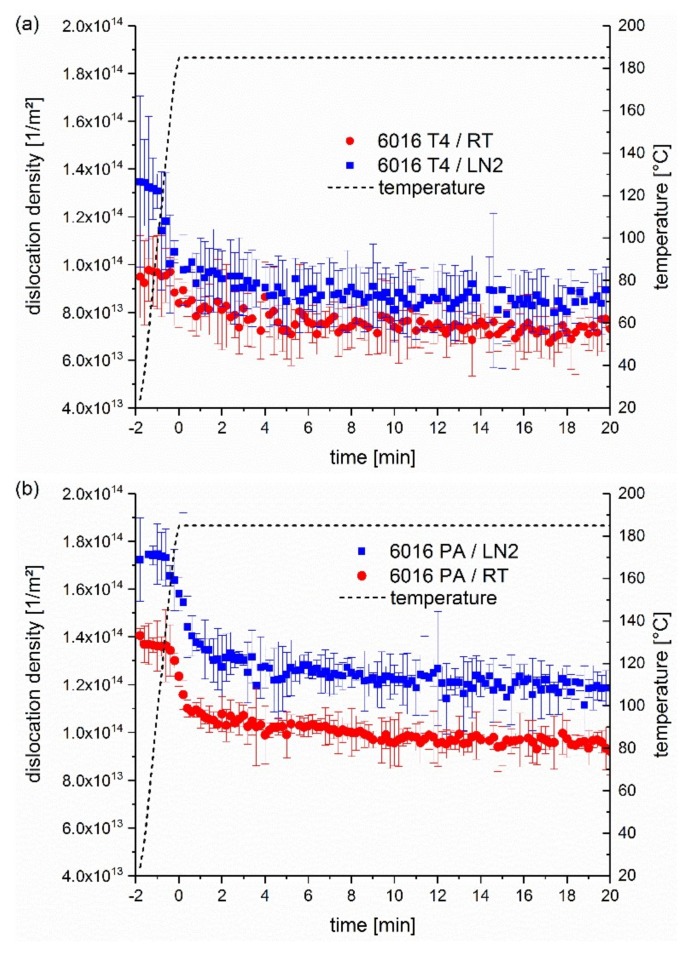
Dislocation densities of (**a**) EN AW 6016 T4 and (**b**) EN AW 6016 PA after 10% of deformation at room temperature and cryogenic temperatures within heating up to 185 °C and 20 min of bake hardening.

In Figure 6, a comparison of dislocation densities before and during the heat treatment of 20 min at 185 °C after straining to 10% is displayed. Note that the beginning of the heat treatment starts only when reaching 185 °C. The dislocation densities are generally greater in the cryogenically pre-deformed samples. This verifies the greater yield strength (Figure 3, Figure 4 and Figure 5) and hardness in Figure 1 of the previous results. During heating up and in the first 5 min of heat treatment, the reduction of dislocation density occurs very fast, and afterwards at a reduced rate, which indicates that the ramp-speed strongly influences the dislocation density’s ability to withstand the artificial ageing treatment.

The sample EN AW 6016 T4 (Figure 6a) shows slightly higher dislocation densities in the first few minutes of the paint bake process than the RT deformed samples, which reduce following the heat treatment. However, this is can only be observed by trend and is near to the scatter of the measurement. In contrast, sample EN AW 6016 PA in Figure 6b shows, throughout the measurement, a significantly greater dislocation density at LN_2_ deformation. This difference is greatest in the first few minutes of the heat treatment and remains approximately constant from 5 min onward, while the dislocation densities slightly decrease.

To compare the synchrotron measurements with the tensile tests, a rough estimation of the dislocation density (ρ) with respect to stress (σ) can be obtained from the Taylor Equation (1) [32]. It includes the following terms: the initial yield stress (σ_0_), a material constant (α = 0.6), the shear modulus (μ = 2.5 × 10^4^ MPa), the Taylor factor (M = 3.06) and the Burgers vector (b = 2.8 × 10^−10^ m) [33].

(1) $$\sigma =\sigma_{0}+\alpha \mu Mb\sqrt{\rho }$$

(2) $$\Delta \sigma =\alpha \mu Mb*\Delta \sqrt{\rho }$$

If only the impact of dislocation density is considered, Equation (2) leads to Δσ of ~7 MPa for alloy EN AW 6016 T4 and to Δσ of ~12 MPa for alloy EN AW 6016 PA after 20 min of artificial ageing.

This difference in stress matches very well with the results of ~8 MPa and 10–15 MPa from the tensile tests in Figure 4. It leads to the conclusion that the difference in strength and hardness of RT and LN_2_ deformed samples is mainly due to a greater dislocation density of the samples deformed in cryogenic conditions, which is maintained during the paint bake process. Therefore, it is confirmed that mechanism (i) plays an important role, which assumes that the dislocation densities are greater at the beginning of the heat treatment and remain so, to a certain extent, for up to 30 min at 185 °C, which could be approved by the synchrotron measurements.

In the naturally aged and pre-aged samples, the dislocation density as well as the ageing kinetics increases through cryogenic pre-deformation compared to pre-deformation. The relation of these two mechanisms is on the side of the maintenance of greater dislocation densities during heat treatment. Above all, alloy EN AW 6016 PA shows a significantly greater difference in hardness, strength and dislocation density between RT and LN_2_ deformation during the first 30 min of artificial ageing. This could be explained by the clusters formed upon pre-ageing, which may stabilize the dislocations structure via coherency strengthening effects [34], and thus restrain recovery. The clusters formed upon pre-ageing represent greater obstacles to dislocations than those formed upon natural ageing, and, more importantly, they are stable and hardly dissolve upon artificial ageing [35].

## 4. Conclusions

In this study, the effects of cryogenic deformation on subsequent artificial ageing were investigated. Naturally aged (T4) and pre-aged (PA) samples of alloy EN AW 6016 were strained to 5%, 10% and 20% at room temperature and under liquid nitrogen, and afterwards aged at 185 °C, for different durations. 

Hardness measurements and tensile tests display an acceleration of artificial ageing kinetics after pre-deformation at RT, as already known from various studies [12,13,14,15,16,17,18,19,20]. In addition, samples deformed at low temperatures show a further increase in strength and hardness, compared to the RT samples subjected to an equal magnitude of strain. This difference in properties between pre-deformation temperatures increases as the degree of pre-deformation increases and is slowly reduced during artificial ageing. The difference could be attributed to two different mechanisms: the greater dislocation density, which is achieved by deformation at low temperatures, and the accelerated hardening kinetics. The pre-aged samples showed a particularly slow decrease of this difference between samples pre-deformed at different temperatures, which still shows a variation in strength of 10–15 MPa between RT and LN_2_ pre-deformation after the full paint bake treatment. Synchrotron measurements revealed that this behaviour could be linked to the greater dislocation density, which is not completely recovered even after prolonged exposure to a temperature of 185 °C and stays at greater values in the case of cryogenic pre-deformation. The results led to a deeper understanding of the industrial paint bake process of cryogenically deformed samples.
